# Mania Induced by Bitter Melon Consumption: A Case Report

**DOI:** 10.1192/j.eurpsy.2026.11207

**Published:** 2026-07-31

**Authors:** B. Ertekin, S. Uygun, O. Citak Ekici, H. Kaya, E. Goka

**Affiliations:** Psychiatry, Ankara Bilkent City Hospital, Ankara, Türkiye

## Abstract

**Introduction:**

Herbal supplements are increasingly used worldwide for various health conditions. However, some herbal products may trigger psychiatric symptoms. Momordica charantia (bitter melon) is a plant widely used in traditional medicine and there are studies showing its potential antidepressant and anxiolytic effects.

**Objectives:**

The aim of this case report is to draw attention to the potential psychiatric side effects of Momordica charantia (bitter melon) and to discuss a manic episode induced by its consumption, which has not previously been reported in the literature. In addition, it aims to emphasize the mental health risks associated with uncontrolled use of herbal supplements.

**Methods:**

In this case report, a detailed clinical evaluation was conducted of a 64-year-old male patient with no psychiatric history who presented with acute-onset manic symptoms following the use of bitter melon.

**Results:**

A 64-year-old male, teacher, married, with three children, presented to the emergency department with insomnia, increased energy, excessive speech, irritability, increased spending, suspiciousness and thoughts of being harmed for the past week. He had no personal or family history of psychiatric illness. Also, no prior hypomanic or manic episodes were reported.

He was admitted to the ward for differential diagnosis and treatment. Upon further evaluation, he reported consuming a herbal supplement containing 2 g/day of bitter melon for one week before symptom onset. His symptoms began after starting the supplement and progressively worsened.

Mental status examination revealed pressured speech, increased speech rate and quantity, loose associations, persecutory and grandiose delusions, and increased psychomotor activity. Physical examination and laboratory tests showed no abnormalities. Brain CT, EEG, and MRI were unremarkable.

At admission, his Young Mania Rating Scale(YMRS) score was 32. He was started on quetiapine XR 800 mg/day, haloperidol 5 mg/day, and valproate 500 mg/day. During follow-up, his speech pressure and quantity decreased, paranoid thoughts regressed, and medication adherence improved. Before discharge, his YMRS score was 8.

Informed consent was obtained from the patient for this case report.

**Conclusions:**

When the literature was reviewed, no case of hypomania or mania secondary to the use of bitter melon was found. This case highlights the potential psychiatric risks of uncontrolled herbal supplement use and presents an unique example of mania triggered by bitter melon. In individuals without a psychiatric history, sudden-onset affective symptoms should prompt an evaluation of herbal supplement use.

**Disclosure of Interest:**

None Declared

